# Supplementary material for: EnzML: multi-label prediction of enzyme classes using InterPro signatures
Source: BMC Bioinformatics. 2012 Apr 25;13:61. doi: 10.1186/1471-2105-13-61 (PMC3483700; doi:10.1186/1471-2105-13-61)
Supplement: Addtional file 5 — The Java code to format the data files, evaluate and predict. The file enzml_java_code.tar.gz contains the Java code used to format database data to ARFF and XML formats, to execute cross and train-test (jackknife) evaluations and to record evaluation results to database. More information is included in the readme.txt file and the Javadoc files. The code can be used with a MySQL database. To use a different database software, other JDBC drivers might be required. [file 1471-2105-13-61-S5.gz › java_code/enzml2011/doc/uk/ac/ed/inf/enzml/test/dataharness/CreateDataTable.html]

CreateDataTable


---


|  |  |  |
| --- | --- | --- |
| **Overview** \| **Package** \| **Class** \| **Use** \| **Tree** \| **Deprecated** \| **Index** \| **Help** | |  |
| **PREV CLASS**   **NEXT CLASS** | **FRAMES**    **NO FRAMES**     **All Classes** |  |
| SUMMARY: NESTED \| FIELD \| CONSTR \| METHOD | DETAIL: FIELD \| CONSTR \| METHOD |  |


---


## uk.ac.ed.inf.enzml.test.dataharness Class CreateDataTable

```
java.lang.Object
  junit.framework.Assert
      junit.framework.TestCase
          uk.ac.ed.inf.enzml.test.dataharness.CreateDataTable
```

**All Implemented Interfaces:**: junit.framework.Test

**Direct Known Subclasses:**: DataTableOneTest, DataTableTwoTest

---

``` public class CreateDataTable extends junit.framework.TestCase ```

Create a table for test instances (and their attributes and classes)

---

| **Field Summary** | |
| --- | --- |
| `static java.lang.String` | `ATTRIBUTE_FIELD` |
| `static java.lang.String` | `CLASS_FIELD` |
| `static java.lang.String` | `INSTANCE_FIELD` |


**Constructor Summary**

`CreateDataTable()`


| **Method Summary** | |
| --- | --- |
| `void` | `createTestTable(java.lang.String tableName)` |
| `java.lang.String` | `getInsertDataSqlStatement()` |
| `java.lang.String` | `getTableName()` |
| `uk.ac.ed.inf.utils.database.TableReader` | `getTableReader()` |
| `void` | `initialise(java.lang.String tableName)` |
| `void` | `setTableName(java.lang.String tableName)` |
| `void` | `testTableInstanceAttribute(java.lang.String instance, java.lang.String attribute)` |
| `void` | `testTableInstanceClass(java.lang.String instance, java.lang.String classLabel)` |

**Methods inherited from class junit.framework.TestCase**

`countTestCases, getName, run, run, runBare, setName, toString`

**Methods inherited from class junit.framework.Assert**

`assertEquals, assertEquals, assertEquals, assertEquals, assertEquals, assertEquals, assertEquals, assertEquals, assertEquals, assertEquals, assertEquals, assertEquals, assertEquals, assertEquals, assertEquals, assertEquals, assertEquals, assertEquals, assertEquals, assertEquals, assertFalse, assertFalse, assertNotNull, assertNotNull, assertNotSame, assertNotSame, assertNull, assertNull, assertSame, assertSame, assertTrue, assertTrue, fail, fail, failNotEquals, failNotSame, failSame, format`

**Methods inherited from class java.lang.Object**

`equals, getClass, hashCode, notify, notifyAll, wait, wait, wait`

**Field Detail**

### INSTANCE\_FIELD

```
public static java.lang.String INSTANCE_FIELD
```

---


### ATTRIBUTE\_FIELD

```
public static java.lang.String ATTRIBUTE_FIELD
```

---


### CLASS\_FIELD

```
public static java.lang.String CLASS_FIELD
```


**Constructor Detail**

### CreateDataTable

```
public CreateDataTable()
```


**Method Detail**

### createTestTable

```
public void createTestTable(java.lang.String tableName)
```

---


### getInsertDataSqlStatement

```
public java.lang.String getInsertDataSqlStatement()
```

---


### getTableName

```
public java.lang.String getTableName()
```

---


### getTableReader

```
public uk.ac.ed.inf.utils.database.TableReader getTableReader()
```

---


### initialise

```
public void initialise(java.lang.String tableName)
```

---


### setTableName

```
public void setTableName(java.lang.String tableName)
```

---


### testTableInstanceAttribute

```
public void testTableInstanceAttribute(java.lang.String instance,
                                       java.lang.String attribute)
```

---


### testTableInstanceClass

```
public void testTableInstanceClass(java.lang.String instance,
                                   java.lang.String classLabel)
```


---


|  |  |  |
| --- | --- | --- |
| **Overview** \| **Package** \| **Class** \| **Use** \| **Tree** \| **Deprecated** \| **Index** \| **Help** | |  |
| **PREV CLASS**   **NEXT CLASS** | **FRAMES**    **NO FRAMES**     **All Classes** |  |
| SUMMARY: NESTED \| FIELD \| CONSTR \| METHOD | DETAIL: FIELD \| CONSTR \| METHOD |  |


---
